# Seasonality, food security, diet quality and nutritional status in urban poor adolescents in Malaysia

**DOI:** 10.1038/s41598-023-42394-6

**Published:** 2023-09-12

**Authors:** Janice Ee Fang Tay, Serene En Hui Tung, Satvinder Kaur, Wan Ying Gan, Nik Norasma Che’Ya, Choon Hui Tan

**Affiliations:** 1 Department of Food Science and Nutrition, Faculty of Applied Sciences, UCSI University, 56000 Kuala Lumpur, Malaysia; 2 Division of Nutrition and Dietetics, School of Health Sciences, International Medical University, 57000 Kuala Lumpur, Malaysia; 3 Department of Nutrition, Faculty of Medicine and Health Sciences, Universiti Putra Malaysia, 43400 Serdang, Selangor Malaysia; 4 Department of Agriculture Technology, Faculty of Agriculture, Universiti Putra Malaysia, 43400 Serdang, Selangor Malaysia

**Keywords:** Climate-change impacts, Socioeconomic scenarios

## Abstract

Seasonality was shown to have an effect on food availability and accessibility, increasing the risk of food insecurity and causing poor diet quality and malnutrition. Therefore, this study aimed to determine seasonal effects on household food security status, diet quality, and nutritional status of urban poor adolescents in Malaysia. A cohort study was conducted among 164 adolescents aged 10–17 from 12 People Housing Programme in Kuala Lumpur, Malaysia during the Northeast (November 2021 till March 2022) and Southwest (June 2022 till September 2022) monsoon. Household food security status was measured using the 18-item USDA Household Food Security Survey Module. Dietary intake was determined using a two-days 24-h dietary recall and translated into Standardized Malaysian Healthy Eating Index (S-MHEI). Anthropometric and haemoglobin level measurements were performed to determine nutritional status. Seasonality was found to have a significant effect on overall diet quality (*p* = 0.021), food groups such as fish (*p* < 0.001), meat/poultry/eggs (*p* = 0.003), and legumes/nuts (*p* < 0.001), and fat nutrient (*p* = 0.037) as well as anaemia status (*p* = 0.020) after controlling the confounders. Although food security did not vary with seasons, seasonality affected the consumption of certain food groups as well as anaemia status for urban poor adolescents. Seasonally sensitive nutrition initiatives should be developed to ensure diet adherence to recommendations, ultimately enhancing the diet quality of urban poor adolescents.

## Introduction

Malnutrition in all forms remain a pervasive global issue that has yet to be fully resolved even with multiple actions by multiple sectors aimed to eradicate the problem. Almost every country in the world continues to be affected by at least a single burden of malnutrition. While anyone can suffer from malnutrition, adolescents are especially vulnerable because of their unique nutritional needs, risks, and eating behaviours as this is the period of rapid increase in both biological and psychological growth and development^1^. Malnutrition appears to be a matter of concern to adolescents in Malaysia^2^. The prevalence of stunting has decreased over the last decade, but the prevalence of thinness, overweight, and obesity has increased^3^, which is not on track to meet the Sustainable Development Goals (UN SDGs) and National Plan of Action for Nutrition in Malaysia (NPANM III) targets. This urges for more considerable progress in tackling the malnutrition problem among Malaysian adolescents. Besides, micronutrient deficiencies among adolescents are also a leading burden of malnutrition, causing global morbidity and mortality^4^. Among all, iron deficiency and iron deficiency-related anaemia account for a majority of adolescents’ disability-adjusted life years (DALY). A national longitudinal cohort study, MyHeART study, suggested an increasing trend of anaemia prevalence from 7.9% to 15.8% across the age at 13 years old to 17 years old among Malaysian adolescents^5^. High anaemia prevalence of 58.4% was reported in a local study among urban poor adolescents^6^. Anaemia is primarily attributed to micronutrient deficiencies which are particularly significant for females^7^. It should be highlighted that the burden of malnutrition can harm adolescents in different ways and these consequences can be associated with the early onset of chronic conditions in adulthood^8–10^.

In view to ensure optimal growth and development is attained during the adolescence period, there is an increasing need to focus on nutritional requirements and diet. The diets of adolescents have been reported as nutritionally poor and fail to meet the dietary guidelines for optimal health and well-being. Poor dietary quality is often characterized by limited diversity of food groups consumed, inadequacy of nutrients, and imbalance in dietary intake^11,12^. This can be observed among Malaysian adolescents who are also the most vulnerable group to have poorer diet quality associated with inadequate consumption of vegetables, milk and dairy products, and fruits as well as excessive consumption of poultry, meat, and eggs^13^. Poverty is also closely linked with the increased intensification of food insecurity levels and poor dietary intake. Urban poor households with low purchasing power prioritize purchasing and consuming staple foods that are perceived as cheaper and affordable but high in calories and less nutrients^14^. This can further worsen poor diet and nutrition outcomes among this population. In addition, a report by UNICEF^15^ revealed that more than 1 in 10 children aged 5 to 17 years living in low-cost flats in Kuala Lumpur have less than three meals a day and 1 in 2 households do not have enough money to buy food in the recent months. This further confirms that food insecurity is a major health-related concern in urban poor communities. Adolescents living in urban poor households who are at risk of food insecurity may be more vulnerable to undesirable and unpredictable consequences such as reduced diet quality, and consequently detrimental health status^8,16–18^.

Emerging research has highlighted the multiple direct and indirect relationships between seasonality with food security, dietary intake, and nutritional status that may occur in different combinations and for varying durations^19–21^. Seasonal variation in food accessibility and availability can also lead to changes in dietary patterns and food utilization, which in turn affects the food quantity and frequency of meals and the variety and quality of the foods people eat^22,23^. A Northern Bangladesh study suggested that insufficient food intake was observed during the summer season^24^ while an Ethiopia study showed a higher prevalence of food insecurity during wet seasons^25^. A neighbouring country study in Cambodia showed a higher intake of rice, starchy roots, vegetables and products, condiments, and spices was observed during wet seasons as compared to dry seasons^26^. A study in Northern Ghana, that looked into seasonality in food consumption, dietary diversity, and quality revealed that the intake of vitamin A-rich fruit and vegetables were higher in rainy seasons while deep yellow, orange, and red vegetables were consumed more in dry seasons^27^. Another study by Egbi et al.^28^ demonstrated the seasonality of nutritional status among Ghanaian school-age children where better height-for-age and BMI-for-age z-score were observed during wet seasons than dry seasons.

In Malaysia's context, the climate is characterized by the Southeast Asia Maritime Continent Monsoon, which consists of the Southwest monsoon (dry monsoon) that occurs from late May to September and the Northeast monsoon (wet monsoon) that occurs from November to March^29,30^. Global warming and climate change are becoming increasingly noticeable in Malaysia and causing rise in temperature, fluctuations in precipitation and rising sea levels^31,32^. These climate variability have substantial impacts on biological and agricultural systems at a range of scales and on human health and nutrition^31^. This impact is further exacerbated by poverty particularly among urban poor communities^31^. Despite the above, to date there is currently no information that provides the understanding on how seasonality affects household food security status, diet quality, and nutritional status in Malaysia. Additionally, climate change, food security, and malnutrition are now commonly regarded as high-priority objectives for many countries in terms of sustainable development, as recognized by the UN SDGs^33^. Therefore, it is of interest to carry out the study to determine seasonal effects on food security, diet quality, and nutritional status of adolescents in urban poor settings in Malaysia.

## Results

### Subject characteristics

The characteristics of the adolescents are presented in Table 1. The mean age of the adolescents was 13.2 ± 2.0 years, predominantly females (51.8%), Malay (63.4%), and receiving secondary education (57.3%) at the point of data collection. The mean monthly household income was MYR 2049.91 ± 1079.54, and the mean household size was 6.08 ± 1.45. The majority of the adolescents were in their advanced/post-pubertal stage (56.7%) and low in physical activity level (64.0%).

**Table 1:** Baseline characteristics of urban poor adolescents (*n* = 164).

	n	%	Mean	SD
Adolescents’ sex				
Male	79	48.2		
Female	85	51.8		
Adolescents’ age (years)			13.2	2.0
Adolescents’ ethnicity				
Malay	104	63.4		
Chinese	8	4.9		
Indian	52	31.7		
Adolescents’ education Level				
Primary School	70	42.7		
Secondary School	94	57.3		
Parental highest educational level				
No formal education	5	3.0		
Primary school	21	12.8		
Secondary school	119	72.6		
Tertiary school	18	11.6		
Parental employment status				
Full time work	101	61.6		
Part time work	18	11.0		
Unemployment/retired	45	27.5		
Parental marital status				
Single	3	1.8		
Married	137	83.5		
Divorced or separated	20	12.2		
Widowed or widower	4	2.4		
Monthly Household Income (MYR)^a^			2049.91	1079.54
Household Size			6.08	1.45
Adolescent’s pubertal status				
Pre/early pubertal	30	18.3		
Mid pubertal	41	25.0		
Advanced/post pubertal	93	56.7		
Physical Activity Level			2.19	0.67
Low	105	64.0		
Moderate	55	33.5		
High	4	2.4		

^a^USD 1 = MYR 4.44 (as of 20 April 2023).

### Seasonal differences in household food security

The subjects had a mean overall HFFS score of 5.46 ± 4.80, with 62.8% experiencing household food insecurity. The prevalence of food insecurity remains similar across seasons, with no significant differences between the Northeast monsoon (63.4%) and Southwest monsoon (62.2%; *p* > 0.05) (Table 2).

**Table 2:** Seasonal differences in household food security, diet quality and nutritional status among urban poor adolescents (*n* = 164).

	Score range	Overall	Seasons		Mean differences	*p*-value
			NW	SW		
Household food security status						
*HFFS score*^*†*^	0–18	5.46 ± 4.80	5.52 ± 5.12	5.35 ± 4.42	0.24 ± 4.72	0.364
Food Insecure^¤^		206 (62.8)	104 (63.4)	102 (62.2)		0.888
Food Secure^¤^		122 (37.2)	60 (36.6)	62 (37.8)		
Diet quality						
*S-MHEI score*^*‡*^	0–100	48.21 ± 10.02	47.85 ± 9.64	48.57 ± 10.39	0.71 ± 12.61	0.469
Poor diet^¤^		197 (60.1)	109 (66.5)	88 (53.7)		0.021*
Diet required improvement		131 (39.9)	55 (33.5)	76 (46.3)		
Good diet		0 (0)	0 (0)	0 (0)		
S-MHEI components score						
Total Grain^†^	0–5	4.66 ± 0.90	4.67 ± 0.77	4.64 ± 1.02	− 0.03 ± 1.08	0.676
Whole Grain^†^	0–5	0.16 ± 0.71	0.17 ± 2.77	0.16 ± 0.73	− 0.01 ± 0.95	0.987
Fruit^†^	0–10	1.36 ± 2.80	1.48 ± 2.77	1.24 ± 2.82	− 0.24 ± 3.92	0.348
Vegetables^†^	0–10	2.84 ± 3.15	2.79 ± 3.28	2.90 ± 3.03	0.11 ± 4.03	0.449
Fish^†^	0–10	3.73 ± 3.93	2.89 ± 3.54	4.56 ± 4.23	1.66 ± 5.04	< 0.001*
Meat, poultry, eggs^†^	0–10	7.85 ± 3.38	7.38 ± 3.57	8.33 ± 3.12	0.95 ± 4.16	0.003*
Legumes, nuts^†^	0–10	2.09 ± 3.47	3.05 ± 4.11	1.13 ± 2.31	− 1.91 ± 4.55	< 0.001*
Milk and milk products^†^	0–10	1.65 ± 2.61	1.33 ± 2.16	1.97 ± 2.96	0.64 ± 3.28	0.019*
Sodium^†^	0–10	6.59 ± 4.50	7.02 ± 4.34	6.15 ± 4.62	− 0.87 ± 6.29	0.040*
Sugar^†^	0–10	9.09 ± 1.67	9.10 ± 1.62	9.07 ± 1.72	− 0.03 ± 2.32	0.853
Fat^†^	0–10	8.20 ± 1.89	7.97 ± 1.85	8.44 ± 1.90	0.71 ± 12.61	0.005*
Nutritional status						
*BMI-for-age*^†^		0.18 ± 1.85	0.23 ± 1.93	0.14 ± 1.76	− 0.09 ± 0.82	< 0.001*
UW^§^		45 (13.7)	22 (13.4)	23 (14.0)		0.013*
NW		162 (49.4)	79 (48.2)	83 (50.6)		
OW		52 (15.9)	24 (14.6)	28 (17.1)		
OB		69 (21.0)	39 (23.8)	30 (18.3)		
*Height-for-age*		− 0.70 ± 1.13	− 0.70 ± 1.11	− 0.70 ± 1.14	0.003 ± 0.34	0.909
Stunting^¤^		35 (10.7)	16 (9.8)	19 (11.6)		0.375
Normal height		293 (89.3)	148 (90.2)	145 (88.4)		
*Haemoglobin level*^†^		12.60 ± 8.40	11.77 ± 1.63	13.13 ± 1.60	0.36 ± 1.39	0.001*
Anaemic^¤^		148 (45.1)	83 (50.6)	65 (39.6)		0.016*
Non-Anaemic		180 (54.9)	81 (49.4)	99 (60.4)		

Seasonal differences were tested using Wilcoxon Signed Ranks Test^†^, Paired T-test^‡^, McNemar test^¤^ and McNemar-Bowker test^§^. Continuous data presented as mean ± SD; categorical data presented as n(%).

### Seasonal differences in diet quality and its components

The mean overall S-MHEI score was 48.21 ± 10.02, which is categorized as poor diet quality. Despite the slightly higher S-MHEI score observed in the Northeast monsoon (48.57 ± 10.39) as compared to the Southwest monsoon (47.85 ± 9.64), no significant difference was observed (*p* > 0.05). There was a significantly higher prevalence of subjects with poor diet quality during the Northeast monsoon (66.5%) versus the Southwest monsoon (53.7%; *p* = 0.021). As for the components of the food group, seasonal differences were observed across the seasons where fish (4.56 ± 4.23 vs. 2.89 ± 3.54, *p* < 0.001), meat/poultry/eggs (8.33 ± 3.12 vs. 7.38 ± 3.57, *p* = 0.003), milk/milk products (1.97 ± 2.96 vs. 1.33 ± 2.16, *p* = 0.019) were consumed significantly more during Southwest monsoon. On the other hand, legumes/nuts (3.05 ± 4.11 vs. 1.13 ± 2.31, *p* < 0.001) were consumed significantly more during the Northeast monsoon. As for nutrient group components, sodium intake (7.02 ± 4.34 vs. 6.15 ± 4.62, *p* = 0.040) was found significantly lower during the Northeast monsoon while better adherence to optimal fat consumption was observed during the Southwest monsoon (8.44 ± 1.90 vs. 7.97 ± 1.85, *p* = 0.005). Other food groups such as whole grain, fruit, and vegetable consumption were notably low across both seasons (Table 2).

### Seasonal differences in nutritional status

Based on the WHO classification, 13.7%, 15.9% and 21.0% of adolescents were underweight, overweight, and obese, respectively. Meanwhile, about 61.7% of adolescents were stunted. The study subjects had mean BAZ as 0.23 ± 1.93 and 0.14 ± 1.76 in the Northeast and Southwest monsoon, which significantly differed across seasons (*p* < 0.001). There was a significantly higher prevalence of obese adolescents during the Northeast monsoon (23.8%) as compared to the Southwest monsoon (18.3%) (*p* = 0.013). The prevalence of stunting was similar across seasons with no significant difference between the Northeast monsoon (9.8%) and Southwest monsoon (11.6%) (p > 0.05). In terms of haemoglobin level, more than half of the adolescents were non-anaemic with a mean haemoglobin level of 12.60 ± 8.40 g/dL and significantly higher during Southwest monsoon compared to Northwest monsoon (13.42 ± 11.73 g/dL vs. 11.77 ± 1.63 g/dL) (*p* = 0.001). Significant difference in the prevalence of anaemic adolescents was observed whereby higher prevalence during Northeast monsoon (50.6%; male 36.1% vs. female 63.9%) as compared to Southwest Monsoon (39.6%; male 43.1% vs. female 56.9%) (*p* = 0.016).

### Seasonal effects on household food security, diet quality, and nutritional status

Table 3 demonstrated the seasonal effects on household food security, diet quality, and status in a Generalized Linear Mixed Model after adjusting for covariates which include adolescent’s sex, age, ethnicity, educational level and pubertal status, parental highest educational level, employment status, marital status, monthly household income, household size, and adolescent’s physical activity level. Seasonality was found to have a significant effect on overall diet quality (B(SE) = 0.58 (0.25), Wald *X*^*2*^ = 5.37, *p* = 0.021), food groups such as fish (B(SE) = -1.77 (0.42), Wald *X*^*2*^ = 17.77, *p* < 0.001), meat/poultry/eggs (B(SE) = -1.06 (0.35), Wald *X*^*2*^ = 9.02, *p* = 0.003), legumes/nuts (B(SE) = 2.14 (0.37), Wald *X*^*2*^ = 34.16, *p* < 0.001) and fat nutrient (B(SE) = -0.43 (0.21), Wald *X*^*2*^ = 4.36, *p* = 0.037). In terms of the nutritional status of adolescents, a seasonal effect was found on anaemia status (B(SE) = 0.60 (0.26), Wald *X*^*2*^ = 5.44, *p* = 0.020). No significant seasonal effects were observed among other variables (*p* > 0.05).

**Table 3:** Seasonal effects on household food security, diet quality and nutritional status among urban poor adolescents (*n* = 164).

	B(SE)	95% CI	Wald Chi-Square	*p*-value
Household Food Security Status				
HFFS score	0.01 (0.11)	– 0.22, 0.23	0.004	0.948
Food Insecure	0.01 (0.26)	– 0.50, 0.51	0.001	0.976
Food Secure				
Diet Quality				
S-MHEI score	– 0.12 (1.06)	– 2.19, 1.95	0.012	0.911
S-MHEI category				
Poor diet	0.58 (0.25)	0.09, 1.07	5.369	0.021*
Diet required improvement				
Good diet				
S-MHEI components score				
Total Grain	0.08 (0.02)	– 0.12, 0.27	0.627	0.428
Whole Grain	0.32 (0.08)	– 0.13, 0.17	0.074	0.985
Fruit	0.24 (0.31)	– 0.38, 0.85	0.567	0.451
Vegetables	0.19 (0.34)	– 0.48, 0.88	0.311	0.577
Fish	– 1.77 (0.42)	– 2.59, – 0.94	17.774	< 0.001*
Meat, poultry, eggs	– 1.06 (0.35)	– 1.76, – 0.37	9.021	0.003*
Legumes, nuts	2.14 (0.37)	1.42, 2.86	34.166	< 0.001*
Milk and milk products	– 0.55 (0.28)	– 1.01, 0.01	3.845	0.050
Sodium	0.65 (0.49)	– 0.32, 1.62	1.728	0.189
Sugar	0.04 (0.19)	– 0.33, 0.40	0.040	0.842
Fat	– 0.43 (0.21)	– 0.83, – 0.3	4.362	0.037*
Nutritional status				
BMI-for-age	0.01 (0.19)	– 0.39, 0.37	0.002	0.967
UW^§^	0.07 (0.22)	– 0.36, 0.50	0.099	0.754
NW				
OW				
OB				
Height-for-age	– 1.11 (0.11)	– 0.32, 0.10	0.998	0.318
Stunting^¤^	0.16 (0.43)	– 1.01, 0.69	0.132	0.717
Normal height				
Haemoglobin level	– 1.25 (0.90)	– 3.11, 0.42	2.244	0.134
Anaemic	0.60 (0.26)	– 1.10, – 0.10	5.441	0.020*
Non-Anaemic				

Seasonal effects were tested using Generalized Linear Model. *p < 0.05.

Model was adjusted for covariates including adolescent’s sex, age, ethnicity, educational level and pubertal status, parental highest educational level, employment status, marital status, monthly household income, household size and adolescent’s physical activity level.

## Discussion

This study showed that household food insecurity among urban poor adolescents persists throughout both seasons with similar prevalence of 63.2% and 62.2% for Northeast monsoon and Southwest monsoon, respectively. This finding was inconsistent with Ethiopia study^25^ and Bangladesh study^34^ which suggested the prevalence of food insecurity was the higher during rainy season as compared to the dry season. Despite insignificant differences in food insecurity across seasons, the high prevalence of food insecurity indicated that food insecurity remains to be a significant public health issue in the urban poor settings. Similar findings of higher prevalence of household food insecurity have been reported in previous local studies with similar study settings among low income communities^16,35–37^. The food insecurity observed among urban poor households can be due to their high vulnerability to all the uncertainties that reduces their access to safe, nutritious, and sufficient food for a healthy lifestyle^14^. As a result, they are forced to compromise food quantity and quality^14^. Urban poor households tend to rely on diets high in refined and processed foods, and fewer fresh staples such as fruits and vegetables^1^. According to the Adolescent Health Survey 2017, the prevalence of adolescents being hungry in Kuala Lumpur (5.7%) was found to be higher than the overall national prevalence (3.9%)^38^, reflecting the occurrence of food insecurity as one of the public health problems among adolescents in Kuala Lumpur.

In this study, the diet quality of urban poor adolescents was reported to be poor and none of them have the diet quality that falls under the good diet quality category. This finding was further supported by past studies^16,39,40^ whereby the diet quality of Malaysian adolescents was generally nutritionally poor and does not adhere to the national guidelines. Particularly, most of the adolescents failed to meet the national guidelines for all S-MHEI components except for the component of total fat intake. The consumption of vegetables, fruit, milk, and milk products, fish was below the recommended serving sizes of the national guidelines. This finding was further confirmed by other local studies^16,40^. The recent Adolescent Health Survey 2022^3^ reported that four in five Malaysian adolescents does not consume enough fruits and vegetables on a daily basis. One of the possible explanations was the phenomenon of food deserts and swamps in urban food environment, whereby limited access to affordable nutritious foods and abundance of unhealthy food options can be observed^41^. Therefore, this is seen to be challenges to these vulnerable and disadvantaged adolescents who are living in urban poverty. Their main source of diverse food is informal food vendors which includes energy-dense street and snack foods that are readily available and accessible, which is also an affordable source of desirable and convenient calories^42^. Hence, limited access to nutritious food and poor dietary diversity is often observed, resulting in poor diet quality among this population.

When looking at the diet quality status, a significant difference existed between two seasons, whereby a poor diet quality was observed during the Northeast Monsoon as compared to the Southwest monsoon. This finding was further explained by the significant differences observed in S-MHEI components of fish, meat/poultry/eggs and milk/milk products, legumes/nuts, and sodium intake. These variations in dietary intake could be due to the variation in food availability and accessibility throughout the seasons. These findings were similar to previous studies where seasonal variations in dietary intake, diversity, and quality were observed in Northern Ghana^27^, Ethiopia^25^, and Western Kenya^43^. These countries share similar characterises of climate and geographical location with Malaysia being close to the equatorial line. Despite the similarity in the climate variability of dry and wet seasons throughout the year, variation in the staples can still be observed due to cultural differences. For example, rice is recognised as the main staple food for Malaysians^44^ but not in Sub-Saharan Africa countries including Kenya, Ghana and Ethiopia in which their main staple food is maize, cassava, wheat and mixed teff^45–47^. The seasonal variation in the consumption of milk/milk products and legumes in the present study was also similar to Abizari et al.^27^ where consumption of milk/milk products were significantly higher in the dry season whilst the consumption of legumes and nuts were significantly higher during the rainy season.

In terms of diet diversity scores, the present finding was consistent with the Ethiopia study where a higher mean diet diversity score was found during rainy seasons, indicating a better practice of a diet that compelled with national guidelines during the rainy season^20^. However, the present findings contradicted the findings by both Abizari et al.^27^ and Waswa et al.^43^ where a higher mean dietary diversity score with a significantly higher intake of dark green leafy vegetables and vitamin A-rich fruits and vegetables were observed during the rainy season instead. The observed seasonal differences in fruit and vegetable consumption could be explained by agriculture production activities that are highly climate-dependent, making it not readily available at all times. Low fruits and vegetables supply are often linked with increased food prices and can be a barrier to fruits and vegetables access and consumption. As a result, diet is compromised, which is particularly relevant among the urban poor population^48^. However, this may not apply to this study as vegetable and fruit consumption of adolescents in the present study does not differ between seasons. During the transition period, adolescents gain more eating autonomy which often opt for unhealthy diet with low fruits and vegetables consumption due to personal preferences^49^. Hence, the seasonality on the vegetable and fruit supply does not significantly contribute to their diet.

Findings from the present study highlight that underweight, overweight/obese, stunting, and anaemia exists in this study population, reflecting that malnutrition continues to be a public health burden in Kuala Lumpur, Malaysia. The prevalence of overweight and obesity in present study was higher than a past local study in Negeri Sembilan^50^ and national studies^4,14^. This reflected that urban poverty may possibly contribute to the increase prevalence of overweight and obesity among this population. Urban poor are more likely to confront challenges in adopting healthy lifestyle, which exposes them to long-term nutritional risk^51^. Besides, this study also revealed that about 45.1% of adolescents were anaemic which is much lower than findings in Kelantan (59.6%)^52^ and Kuala Lumpur (58.4%)^6^, but much higher than national prevalence (Malaysian adolescents 15–19 years old) of 20.5%^53^. It should be noted that the Kelantan study was carried out in rural district, where the study setting differed from the present study setting with high urbanization rate. However, both study subjects were from the same low income group (B40) and shared similar socioeconomic background. Therefore, it is still comparable and important to highlight in the present study. In addition, our finding on the anaemia prevalence is comparable to studies conducted in Indonesia (44.0%)^54^. A possible explanation is that adolescents are more susceptible to anaemia due to their rapid increases in physiological growth, sexual, and neurological changes that requires additional nutritional requirements^55^. Poor diet quality and food insecurity as indicated by the results might also be responsible for the existence of multiple forms of malnutrition in this study. While a compromised diet with inadequate nutrients intake will impede the growth and development, forced monotonous diet with high calorie can lead to overweight and obesity^56^. In addition, poor diet quality may contribute to poor intake of iron absorption enhancers and carriers including protein and micronutrients, subsequently resulting in low haemoglobin level^57^.

When comparing between seasons, a higher prevalence of overweight and obesity was observed during the Northwest monsoon compared to the Southwest monsoon. This could be partly explained by the differences in dietary intake and quality between seasons as explained above. In addition, a higher prevalence of anaemia was observed in Northeast monsoon compared to Southwest monsoon, which coincides with the decrease in the intake of iron-rich food such as meat/poultry/eggs and fish during this season as indicated in the results. This can be due to the climate variability of unprecedented major floods during Northeast monsoon that affected the production and distribution of livestock and food supplies^58^. Nonetheless, seasonal variation in haemoglobin level and anaemia status was not observed in the Ghana study by Egbi et al.^28^. This could be due to the differences in the study setting and population as the study in Ghana focuses on school age children in peri-urban communities instead.

When assessing seasonal effects using the Generalized Linear Mixed Model, seasonal effects were observed on diet quality status, S-HMEI components such as fish, meat/poultry/eggs, and legumes/nuts, as well as anaemia status after confounders: adolescent’s sex, age, ethnicity, educational level and pubertal, parental highest educational level, employment status, marital status, monthly household income, and household size were taken into consideration. Several studies have shown seasonal effects on dietary intake and diversity^25–27,43,59^ as well as nutritional status^25,28,34^, however, the pattern of seasonal fluctuation on dietary intake and nutritional status were shown to be inconsistent. The seasonal effects in diet quality and certain food groups influenced the seasonal effects in the prevalence of obesity and anaemia. Given that no seasonality on household food security was observed in this study, this result implies that variations in dietary quality and its components could be due the changes in either food costs or food availability of certain food source that contribute to the shift in the dietary intake and diversity^32^. Nevertheless, due to the study sample size, this study findings would need to be interpreted with caution. Despite inconsistencies in the findings, seasonality remains to be one of the elements to be considered in dietary intake and nutritional status considering how seasonality is an indication of climate variability which has been shown to have significant implications to food security, human nutrition and health^60,61^. This information serves as the basis for the establishment of various strategies and climate-sensitive policies to mitigate and address seasonal effects on food insecurity and malnutrition among urban poor communities.

This study is not without limitations. The study results may not be generalizable to other populations as the study focused on urban poor adolescents in Kuala Lumpur, Malaysia. As dietary intake and quality was assessed using a 2-day 24-h dietary recall, it may not be able to reflect the adolescent’s habitual dietary intake especially on weekdays during each season due to recall bias. Besides, additional effects of coronavirus disease 2019 including food security status, financial status, and dietary intake were not assessed suggesting further investigation is needed. The insignificant findings in the seasonal effects on household food security and nutritional status could also be attributed to the short study period and small sample size. With this, longitudinal studies with larger sample size and longer study durations of more than two consecutive years with regular follow-ups could be more informative and better reflect the seasonal effects. Future research may also consider expanding the study to urban areas of Malaysia’s East Coast (eg. Kelantan, Terengganu and Pahang) that is often affected by seasonality, which could provide a more comprehensive understanding of the seasonal effects at a national level. Despite these limitations, this study provides valuable information and contributes to the existing, yet limited literature on the seasonal effects on household food security, diet quality, and nutritional status of urban poor adolescents, in which food insecurity and poor diet quality remain appreciable concerns.

In conclusion, seasons had an effect on the consumption of fish, meat/poultry/eggs, and legumes/nuts as well as anaemia status of the urban poor adolescents. Although household food security does not vary with seasons in the present study, household food insecurity remains prevalent among urban poor communities. It is suggested that seasonally sensitive nutrition initiatives should be developed to ensure diet adherence to recommendations, ultimately enhancing the diet quality and nutritional status of urban poor adolescents.

## Methods

### Study design and data collection

This is a prospective cohort study that examined the seasonal variation in food security and nutritional status of the urban poor adolescents. The study protocol was previously described^62^. Subject recruitment was carried out from 12 People’s Housing Programme (*Perumahan Awam, PA* or *Program Perumahan Rakyat, PPR*) located in Kuala Lumpur, Malaysia, using a multistage stratified random sampling method^63^. There is no official definition for urban poor adolescents. For this study, urban poor adolescents are defined as adolescents who stay in PA/PPRs, an initiative by government for resettlement of urban slums to meet the housing needs of the low-income group (B40)^64^. Urban poor adolescents who are under any special diet or diet restriction, have chronic medical problems, are pregnant or lactating, or were infected with COVID-19 at the point of the study were excluded from the study upon recruitment. The subjects were recruited during the Northeast monsoon (November 2021) and followed up during the Southwest monsoon (June 2022) to capture a different season. Out of 663 adolescents who were invited to participate in the study, 279 adolescents consented and completed the data collection at baseline during the Northeast monsoon. Some adolescents were lost to follow-up (n = 115) during Southeast monsoon with an attrition rate of 41.2%. Only adolescents with data at both time points (n = 164) were included in the final analysis.

### Ethics approval and consent to participate

The ethical approval was obtained from the Institutional Ethics Committee of UCSI University in accordance with the Declarations of Helsinki [Reference code.: IEC-2021-FAS-03)] and permission to conduct the study from Kuala Lumpur City Town Hall (DBKL) [Reference code: DBKL/JPKKB/SPZ/29–3 Jld 13 (48)] before the commencement of the study. Parents/guardians provided signed written informed consent to allow their child’s participation, and assent was obtained from adolescents to indicate their willingness to participate. The personal information of the participants will be kept anonymous and confidential.

## Measures

### Sociodemographic characteristics

Sociodemographic data were collected using a self-administrated questionnaire upon recruitment. Adolescent information includes age (in years), sex (male and female), date of birth, ethnicity (Malay, Chinese, Indian, and others), educational level (primary school and secondary school), and household size, whereas parental/guardian information includes, highest educational level (no formal education, primary school, secondary school, tertiary school), marital status (single, married, divorced or separated, widowed or widower), occupation (full-time work, part-time work, unemployment, retired), and monthly household income (in MYR) were collected.

### Pubertal status

Pubertal Development Scale (PDS) was used to assess the pubertal status of adolescents^65,66^. A total of five items are assessed to rate the growth spurt, body hair growth, and skin changes using a four-point scale including not yet started (1), barely started (2), definitely started (3), seems complete (4), and I don’t know (0). On a similar scale, boys will be asked to rate their development of facial hair and voice change, whereas girls rated their breast development and whether they have reached menarche, present (4) and absent (1). Based on the sum of the scores, adolescents will be categorized into pre/early pubertal, mid-pubertal, and advanced/post-pubertal^67^.

### Household food security status

Household food security status was assessed using the United States Department of Agriculture (USDA) 18-item Household Food Security Survey (HFSS) Module^68^ that captures all severity levels of food security and children’s condition in the household for both seasons. This instrument is a widely adopted tool in a variety cultural and linguistic contexts worldwide^69^ and been used in a national survey in Malaysia^70^. This section was filled by the parents or the guardians as parents or guardians will be responsible for food purchasing or preparing for the household and the adolescent. An affirmative response to each question including “yes”, “often true”, “sometimes true,” “almost every month,” and “some months but not every month” were given one point (1) and the household’s raw score was calculated. The household food security status was categorized into high food security (score = 0), marginal food security (score = 1–2), low food security (score = 3–7), and very low food security (score = 8–18). Households with high or marginal food security were categorized as food secure group, while households with low or very low food security were categorized as food insecure group^68^.

### Dietary assessment

A two-day 24-h dietary recall of one weekday and one weekend, was used to determine the dietary intake of subjects for both seasons. Subjects were interviewed and requested to recall and describe the food items and beverages consumed 24 h preceding each of the two surveys. Detailed information including ingredients, brand names of commercial products, food preparation method as well as the portion sizes consumed with the aid of a set of standard household measurements such as spoons, teaspoons or tablespoons, cups, glasses, bowls, and plates, along with the use of the Food Atlas book^71^. Dietary recall data obtained were analyzed using the Nutritionist Pro® Software Version 4.0 (Axxya Systems, Stafford, TX, USA) based on the food listed in the Malaysian Composition Database Nutrient Composition of Malaysian Food. This was then further translated for the diet quality of the subjects using the Standardized Malaysian Healthy Eating Index (S-MHEI)^72^.

The S-MHEI consisted of 11 components, including eight food groups (total grains, whole grains, fruits, vegetables, meat/poultry/eggs, legumes/nuts, and milk/milk products) and three nutrient groups (total fat, sodium, and sugar). The scoring of these components is calculated based on the serving size and nutrient intake recommended by the Malaysian Dietary Guidelines^73^ and Malaysian Dietary Guidelines for Children and Adolescents^74^. The score of each component ranged from 0 (lack of compliance) to 10 (full of compliance), which is calculated proportionately for the in-between whole-number responses. However, as total grains and whole grains are from the same food group, the maximum score provided to each component is five to avoid scoring overlap. The total S-MHEI score was obtained by summing the scores of each component. The composite score in percentage terms was calculated using the formula: (Total Score Obtained from 11 components/10 × 100%). The possible composite score ranged from 0 to 100, in which a higher S-MHEI score indicated better diet quality. The diet quality of subjects was categorized into good diet quality (score =  > 80%), diet quality needs an improvement (score = 51 – 80%), and poor diet quality (score =  < 51%)^72^.

### Anthropometric assessment

The anthropometric measurements of body weight and height of the adolescents were assessed using Bioelectrical Impedance Analyzer (OMRON HBF-375 Karada Scan Body Composition Monitor OMRON HEALTHCARE Co., Kyoto, Japan) and a calibrated vertical stadiometer (Seca 213 portable stadiometer, SECA GmbH & Co., KG. Hamburg, Germany), respectively. Both height and weight measurements were measured twice, and the mean was determined as the final measurement. The nutritional status of adolescents was determined based on Z-scores for height-for-age (HAZ) and body mass index (BMI)-for-age (BAZ) of WHO growth reference 2007 for adolescents between 5 and 19 years old using WHO AnthroPlus version 1.0.3 software (WHO, Geneva, Switzerland). The classification based on WHO growth reference charts as follows, stunting (HAZ < -2SD), normal height (HAZ > -2SD), thinness (BAZ < -2SD), normal weight (− 2SD ≤ BAZ ≤  + 1SD), overweight (+ 1SD < BAZ ≤  + 2SD), and obesity (BAZ >  + 2SD)^75^.

### Haemoglobin level test

A haemoglobin level test was performed to identify the anaemia status using the HemoCue haemoglobinometer (HemoCue® Hb 201 + System, Angelholm, Sweden), point-of-care testing on capillary blood samples. Before the blood sample collection, a brief explanation of the blood sampling method and its potential risks was provided. A drop of blood was collected and filled in the HemoCue microcuvette, and the haemoglobin result was shown within 10 min. Anaemia status and level of severity are determined according to the age and gender-specific haemoglobin cut-off point as defined by WHO^76^: (i) < 11.5 g/dL for 5 to 11 years of age; (ii) < 12.0 g/dL for 12 to 14years of age and non-pregnant women (15 years of age and above); (iii) < 13.0 g/dL for men (15 years of age and above).

### Physical activity assessment

Physical activity questionnaire for older children (PAQ-C) was used to assess the physical activity level^77^. The questionnaire consisted of 10 items to assess physical activity level of subjects over the past seven days using s five-point scale ranging from ‘no activity’ scoring one, and ‘7 times or more’ scoring five. The mean PAQ-C score were calculated with range from zero (low physical activity) to five (high physical activity)^77^.

### Statistical analysis

Statistical data analyses were performed using IBM SPSS 25.0 (SPSS Inc., Chicago, IL, USA). A test of normality will be performed using a skewness value (between − 2 and + 2) for the continuous variables involved in this study^78^. The descriptive analysis was used to determine mean and standard deviation (SD) for normality distributed variables, median and interquartile range (IQR) for skewed variables, and percentage and frequencies for categorical variables. A McNemar test and McNemar-Bowker test were performed to determine the seasonal differences in proportions of household food security status, diet quality status, and nutritional status across the seasons. Paired T-test and Wilcoxon signed-rank test were used to test the seasonal differences in the mean or median of household food security, diet quality, and nutritional status across seasons. The Generalized Linear Mixed Model was used to determine the seasonal effects on household food security status, diet quality, and nutritional status. The statistical significance level for all analyses was set at *p* < 0.05.

## Data Availability

The datasets used and analysed during the current study are available from JEF Tay (janicetay96@gmail.com) upon reasonable request.
